# Therapeutic Reciprocity in Precision Neuro-Oncology: An Ethical Threshold for Molecular Stratification in Low- and Middle-Income Countries

**DOI:** 10.7759/cureus.115729

**Published:** 2026-09-03

**Authors:** Maryem Tanweer, Muhammad Bilal Yousaf, Jazalah Atif, Haseeb Mehmood Qadri, Zia-Ul-Rehman Najeeb

**Affiliations:** 1 Neurological Surgery, Punjab Institute of Neurosciences, Lahore, PAK; 2 Medical School, Fatima Memorial Hospital College of Medicine and Dentistry, Lahore, PAK; 3 General Surgery, Lahore General Hospital, Lahore, PAK

**Keywords:** brain tumors, central nervous system, developed countries, developing countries, genomics, low- to middle-income countries, neoplasms, neuro-oncology, world health organization

## Abstract

Central nervous system (CNS) tumours are one of the most important entities in neurosurgery. The recent changes in the framework of the World Health Organization (WHO) classification of tumours reshape the diagnostic paradigm from morphological assessment to molecular characterization. This has elevated the standards of diagnostic precision, as well as in tailoring targeted regimes for CNS tumour management. The problem, however, lies in its limited applicability in low- and middle-income countries (LMICs) as opposed to high-income countries (HICs), where greater diagnostic precision is more likely to translate into improved patient outcomes through access to appropriate targeted therapies. Collectively, these challenges have led to the emergence of the concept of "therapeutic reciprocity." This principle emphasizes that improvements in diagnostic precision do not necessarily translate into equitable access to targeted therapies, particularly in LMICs. Consequently, without a realistic prospect of improving treatment availability, patient outcomes, or follow-up care, these out-of-pocket and non-actionable tests raise ethical and clinical-value concerns in LMIC settings. However, diagnostic precision upholds its own independent value for prognostication, avoidance of unnecessary toxicities, medication selection, surgical planning, and eligibility for clinical trials. LMICs play a huge role in collecting genomic datasets through sample donations and collaborations, yet remain underprivileged in therapeutic innovations reaching their population. Therefore, this editorial highlights these concerns while proposing strategies to bridge the gap in diagnostic precision and therapeutic innovations between LMICs and HICs.

## Editorial

Central nervous system (CNS) tumours are one of the top 10 neurosurgical conditions for which neurosurgical care is deemed essential, as adequate, timely, and effective interventions leave a remarkable lifelong impact [1]. In 2021, the World Health Organisation (WHO) published a framework for CNS tumour classification that redefined the diagnostic landscape, shifting from traditional morphological assessment to molecular identity [2]. Precision oncology represents the transition from conventional, uniform tumour treatment towards individualised therapeutic strategies guided by genomic and molecular characterisation. In neuro-oncology, this genetic profiling transformed the management of CNS malignancies by incorporating molecular biomarkers. Precision neuro-oncology now extends beyond prognostic refinement: it reframes a tumour's identity. However, this genetic profiling of tumours introduces an ethical challenge when the remarkable evolution of molecular diagnostics has not been paralleled by equivalent advances in targeted therapeutic strategies in low- and middle-income countries (LMICs) due to unbearable financial strain [1,3]. To better understand this fundamental transformation, gliomas serve as a representative example. Gliomas, the most common malignant CNS tumours, are divided into molecular subtypes based on IDH mutation, 1p/19q codeletion, telomerase reverse transcriptase (TERT) promoter mutation, epidermal growth factor receptor (EGFR) gene amplification, and chromosome 7 gain combined with chromosome 10 loss [4]. These molecular biomarkers are central for accurate preoperative, intraoperative, and postoperative evaluations, as well as targeted treatment regimens, including antiangiogenic therapies, IDH inhibitors, BRAF/MEK inhibitors, FGFR and TRK inhibitors, and CDK4/6 inhibitors [3]. Thus, if genomics does not transform into accessible therapeutic interventions, its routine application in LMICs questions the ethical justification for using these highly precise investigations only for diagnostic purposes. This challenge leads to the introduction of the concept of 'therapeutic reciprocity'.

Therapeutic reciprocity emphasises that improvements in diagnostic precision do not necessarily translate into equitable access to targeted therapies, particularly in LMICs. Consequently, without a realistic prospect of improving treatment availability or patient outcomes, this non-actionable and out-of-pocket molecular stratification may raise ethical and clinical concerns in LMIC settings. LMICs play a huge role in collecting genomic datasets through sample donations and collaborations, yet remain underprivileged in therapeutic innovations reaching their population. Therefore, this editorial highlights these concerns while proposing strategies to bridge the gap in diagnostic precision and therapeutic innovations between LMICs and HICs.

Knowledge without agency: a clinical microcase

Consider a hypothetical case of a 25-year-old woman presenting with focal seizures and headaches. Imaging and molecular testing identified an IDH-1-mutant astrocytoma with favourable prognostic markers. While genomic analysis enabled precise diagnosis and suggested a targeted treatment approach involving radiotherapy, temozolomide, and vorasidenib, these therapies were largely inaccessible due to financial and structural barriers. The resulting gap between detailed prognostic knowledge and limited treatment options caused her significant psychological distress, including anxiety, depression, and feelings of abandonment. This case highlights how precision diagnostics without accompanying therapeutic access may deepen patient suffering rather than alleviate it. This situation strengthens the hypothesis that extensive molecular profiling in the context of contemporary precision neuro-oncology, but a lack of suitable target regimes often deepens patients' suffering [5].

Cognitive vulnerability and relational consent

Brain tumour patients frequently experience cognitive and behavioural impairments affecting executive function, personality, memory, decision-making capacity, and a wide spectrum of defects depending upon the location, size, and mass of these tumours [6,7]. Such vulnerabilities challenge the adequacy of traditional autonomy-based informed consent models, as patients face difficulty making complex prognostic decisions on their own, especially in contexts with limited genetic counselling and neuropsychological support. Opposite to this, another more supportive framework, a relational consent model, has developed, which incorporates family involvement in the process of decision-making for the patient's treatment while preserving the patient's privacy. Thus, this model creates an environment of prognostic discussions rather than one-sided treatment briefings and develops a social support framework for active decision-making with respect to the patient's compromised status. All of these favour a relational consent model, a better version than that of the outdated informed consent, especially in LMICs.

While LMICs contribute increasingly to global genomic datasets through tumour sample donations and collaborations, therapeutic advances largely remain confined to high-income countries. Despite this substantial output, LMICs remain underprivileged and less represented as compared to high-income countries (HICs) when it comes to high-impact, citation-rich research and therapeutic innovations reaching their population [8]. This asymmetry denotes a form of genomic extractivism, where molecular data is extracted without reciprocal infrastructural or therapeutic benefits returning to the source communities. Ethical therapeutic reciprocity must extend beyond clinical care to encompass international research partnerships, ensuring enforceable benefit-sharing and capacity-building commitments that promote global equity.

In LMICs, rapid adoption of molecular diagnostics in the absence of essential supports, such as tumour boards, neuropathology expertise, regulatory oversight, and trained workforce, creates ethically fragile healthcare ecosystems. A structured threshold model is needed, scaling molecular stratification only when systems meet minimal standards for therapeutic access, interpretive expertise, and follow-up care. Thereby, we propose a three-tier framework for operationalising our core concept of therapeutic reciprocity in molecular stratification and for a practical implication of this threshold model (Table 1).

**Table 1:** Author-proposed three-tier framework for operationalizing the core concept of therapeutic reciprocity.

Tiers	Diagnostic modalities	Infrastructure and requirements	Indication for moving to the next level
Tier 1 (Entry level)	Conventional histopathology (IDH1-R132H, ATRX, p53) and low-cost immunohistochemistry (IHC)	Histopathology laboratories already established in many distinct-level hospital and clinical settings	Ambiguous results, suspecting false negatives/positives, atypical IDH mutations, diagnostic discordance warranting Tier 2 evaluation
Tier 2 (Intermediate level)	Molecular assay including Co-deletion analysis (1p/19q) through FISH, PCR, MGMT methylation testing	Clinical and laboratory settings at the regional level registered under a cancer directory and regulated by a multidisciplinary tumour board directly accountable to the national level	Requirement for targeted therapy selection, clinical trial eligibility screening, or unresolved diagnostic ambiguity following Tier 1 and Tier 2 testing
Tier 3 (Final level)	Advanced Next-Generation Sequencing (NGS) profiling	Advanced research centres affiliated with well-resourced institutions or funding bodies to ensure therapeutic reciprocity between trial participation and patient care access	Highest tier

We suggest that progression between the tiers outlined in Table 1 should be guided by objective parameters, including diagnostic concordance, access to certain therapeutic and clinical trials matching diagnostic yields, and mutually befitting the patients. Furthermore, the advancement between these tiers must be ensured in the presence of a certified tumour board at the regional level, capable of interpreting and analysing the results rather than solely relying on technological ambitions. These tumour boards must be registered under a unified national body linking them to the national cancer registry, directly ensuring transparency and the smooth functioning of this framework. This phased approach aligns technological ambition with moral responsibility.

Precision neuro-oncology should be understood as a moral obligation, not merely a scientific pursuit. True precision is achieved not by subdividing tumours into ever finer molecular categories, but by advancing knowledge, equitable access to therapies in LMICs, and patient dignity in tandem. Therapeutic reciprocity should become the guiding ethical standard for integrating genomic innovation into neuro-oncology worldwide, ensuring that scientific progress benefits all patients, regardless of geographic or economic context.
